# 2D fluorescence spectroscopy for real-time aggregation monitoring in upstream processing

**DOI:** 10.1186/1753-6561-7-S6-P94

**Published:** 2013-12-04

**Authors:** Karen Schwab, Friedemann Hesse

**Affiliations:** 1 Institute of Applied Biotechnology, University of Applied Science Biberach, 88400 Germany

## Introduction

Product aggregation is one side effect of rising yields due to process improvement and therefore accompanied with massive product loss during downstream processing (DSP). But it is already in literature described, that product aggregation also occurs during the fermentation process and is caused by various process operations [1]. Real-time bioprocess monitoring and thus on-line product quality control during upstream processing (USP) enables to address this issue during process development. For bioprocess control, 2D fluorescence spectroscopy in combination with chemometric modeling based on fluorescence signals derived from cells and medium components is a promising tool and described in literature [2]. Furthermore extrinsic fluorescence dyes are widely used to detect and quantify aggregated protein [3]. In this study, 2D fluorescence spectroscopy in combination with three different extrinsic fluorescence dyes were evaluated, in order to establish a process control tool which enables real-time product control during USP.

## Materials and methods

A CHO DG44 cell line producing a monoclonal antibody (mAb) was cultivated in a 2 liter bioreactor (Sartorius AG) in fed-batch mode. Metabolites and substrate concentrations were determined using Konelab 20XT (Thermo Scientific) and cell concentration and viability via CEDEX XS system (Innovartis-Roche AG). The product titer was determined with protein-A HPLC. Furthermore, culture supernatant samples were applied to the size exclusion column Yarra S4000 (Phenomenex) after filtration. The intrinsic fluorescence signal at 355nm was recorded with a fluorescence detector (Gynkotek), in order to determine the monomer to aggregate ratio in the sample. Samples were taken twice a day and incubated with ANS, bis-ANS and Thioflavin T at 3 different concentrations respectively. Full 2D scans from 270nm to 590nm of these samples were taken with the DELTA BioView^® ^sensor. These scans were used as data input for chemometric modeling, where the target data was the mAb aggregate concentration.

## Results

**Figure 1:**
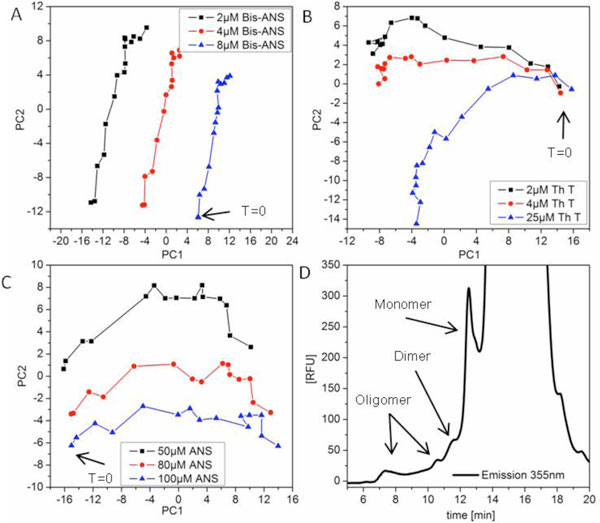
**PCA score plots for all Bis-ANS (A), thiovlavin T (B) and ANS (C) concentrations, where PC2 is displayed over PC1**. T = 0 indicates data of 2D scans taken directly after inoculation. (D) SEC chromatogram of the intrinsic fluorescence emission signal at 355nm. Monomer, dimer and oligomer fractions of mAb were detectable; furthermore a separation from the medium and host cell protein signal was possible.

A common approach to analyze aggregated mAb in cell culture comprises the isolation of the mAb by protein A HPLC subsequently followed by size exclusion chromatography [1,4]. However, the capture step itself may have an influence on product aggregation. Therefore, in this study we tried to avoid the capture step by directly applying cell culture supernatant onto the size exclusion column after a filtration step. The signal derived from the cell culture medium and host cell proteins could be separated from mAb monomer and aggregate signal (Figure 1D). This allowed direct quantification of mAb aggregates in culture broth via size exclusion chromatography (SEC). Fluorescent dyes such as ANS, and its dimeric analogon 4,4'-bis-1-anilinonaphthalene-8-sulfonate (Bis-ANS) as well as thioflavin T interact noncovalently with hydrophobic regions of the aggregated protein [3]. To our knowledge, up to now these dyes were not used as additives in mammalian cell cultures. Therefore, a major concern was their toxicity towards the CHO production cell line. Toxicity screens in microtiter plates (data not shown) revealed that already 4μM bis-ANS as well as 4μM thioflavin T reduced the specific growth rate strongly. The in literature reported concentrations for these dyes in DSP approaches [3] were considerably higher hence their sensitivity limits in cell culture had to be evaluated. In order to enable a direct comparison of fluorescence intensity increase generated by dye aggregate interaction, the DELTA BioView^® ^sensor was used at-line during the fed-batch fermentation. For chemometric modeling, fluorescence maps were preprocessed by principal component analysis (PCA), in order to capture the data input with the highest variance over the cultivation time. PCA results indicated that the sensitivity of Bis-ANS and ANS was very high towards aggregated mAb. Furthermore, increasing Bis-ANS concentrations increased the score values of PC1 in general (Figure 1A), contrary to ANS where score values of PC2 increased (Figure 1C). For thioflavin T score values differed greatly when low and high dye concentrations were compared, starting at one point (Figure 1B). Furthermore, the mAb aggregate titer was used as target for partial least square regression (PLS) (Table 1) and resulting calibration and validation models showed low root square mean error (RMSE) values as well as good slopes and R-squares for ANS and Bis-ANS. Besides that, the chemometric model computed with 2D scans taken from cell culture without additional dye showed a slope of 0.7 and R-square value of 0.72 for the validation data set. This indicated that the quality of the chemometic models seemed to be improved when an additional fluorescence signal based on dye mAb aggregate interaction was generated in the 2D scans. Moreover, only 25μM thioflavin T enabled a solid calibration model (Table 1). This raised the suspicion, that there might be only weak interactions of dye and aggregated mAb. In consequence these preliminary results indicated, that thioflavin T which is normally used for detection of fibrils seemed to be less favorable for the detection of mAb aggregates.

**Table 1:** PLS results for selected dye concentrations used in the fed-batch fermentation experiment.

Dye	PC's		R-Square	RMSE	Offset	Slope
w/o dye	3	calibration data set	0.96	1.27	0.43	0.96
		validation data set	0.72	3.62	2.75	0.70
2μM Bis-ANS	4	calibration data set	0.98	10.9	2.28	0.98
		validation data set	0.93	19.36	-4.20	0.97
80μM ANS	4	calibration data set	0.98	0.82	0.18	0.98
		validation data set	0.85	2.08	1.44	0.85
25μM Th T	2	calibration data set	0.99	5.18	0.52	0.99
		validation data set	0.96	14.54	6.23	0.93

2D fluorescence scans were taken as x-data and the mAb aggregate concentration was used as target data for chemometric modeling. Validation data sets were generated with cross validation.

## Conclusions

Suitable fluorescence dye candidates were selected and based on sensitivity and toxicity, ANS and Bis-ANS proved to be promising candidates for further work. Direct quantification of mAb aggregates in cell culture broth was possible with SE-HPLC based on the intrinsic fluorescence of mAb. The fed-batch fermentation experiment, where the DELTA BioView^® ^sensor was used at-line, enabled a direct comparison of different dye concentrations. Therefore, this experiment demonstrated that for bis-ANS even lower concentrations than already used might be applicable due to its high sensitivity towards mAb aggregates. Moreover, the results indicated that product aggregation is not only a side effect of rising titers, because mAb aggregates were also present at early fermentations time points.
